# Experimental Model for Sutureless Proximal Anastomosis by the Viabahn Open Revascularization TEChnique (VORTEC)

**DOI:** 10.5935/1678-9741.20160087

**Published:** 2016

**Authors:** Lucas Marcelo Dias Freire, Giuliana Biasi Gobbi, Inácio Maria Dal Fabbro, Fábio Hüsemann Menezes

**Affiliations:** 1 Faculdade de Ciências Médicas da Universidade Estadual de Campinas (FCM-UNICAMP), Campinas, SP, Brazil.; 2 Fundação Centro Médico de Campinas (CMC), Campinas, SP, Brazil.; 3 Faculdade de Engenharia Agrícola da Universidade Estadual de Campinas (FEA- GRI-UNICAMP), Campinas, SP, Brazil.

**Keywords:** Anastomosis, Surgical, Models, Animal, Swine

## Abstract

**Introduction:**

In the treatment of complex aneurysms, debranching is an extra-anatomical revascularization of visceral arteries followed by endograft coverage of the thoracoabdominal aorta. It eliminates the need for a thoracotomy and aortic clamping, but requires the performance of several technically demanding visceral anastomosis. In 2008, Lachat described visceral revascularization with the use of a sutureless distal anastomosis, performed by the telescoping of an endograft in the visceral branch, named VORTEC (Viabahn Open Revascularization TEChnique).

**Objective:**

An experimental model was created to test the feasibility and short term results of performing a telescoped proximal anastomosis to the abdominal aorta.

**Methods:**

Swine model. The abdominal aorta was dissected and ligated between the renal arteries and the iliac vessels. Three centimeters bellow the renal arteries a Viabahn endograft was telescoped for 2 cm into the proximal aorta. The other extremity was conventionally anastomosed to the distal aorta. Patency, sealing and tensile strength of the anastomosis were tested.

**Results:**

Time for performing the telescoped anastomosis was shorter (5.4±2.8 min *versus* 10.3±3.4 min, *P*<0.05). All grafts were patent and both types of anastomosis presented no bleeding. Immediate tensile strength showed a higher strength of the conventional suture (22.7 x 14.3 N, *P*<0.09). After 30 days there was no pseudo-aneurysms and the strength of the conventional and VORTEC anastomosis were similar (37.3 x 40.8 N, respectively, *P*=0.17).

**Conclusion:**

Telescoped proximal anastomosis by the technique of VORTEC is feasible. After 30 days the tensile strength of the both anastomosis were similar.

**Table:** 

**Abbreviations, acronyms & symbols**	
EPTFE	= Expanded polytetrafluoroethylene graft
N	= Newton
VORTEC	= ViaBahn Open Revascularization TEChnique

## INTRODUCTION

Conventional vascular anastomosis requires extensile exposure, circumferential dissection and temporary occlusion of the vessels. This technique was initially described by Alexis Carrel, in 1902^[^^1^^]^, and despite several technical improvements it has remained basically the same.

Several devices for sutureless anastomosis were developed since that time, as grafts with rings^[^^2^^]^, connectors^[^^3^^]^, clips^[^^4^^]^ and even magnets^[^^5^^]^.

In complex aortic surgeries, the time to perform an anastomosis is related to ischemic and reperfusion injuries and can lead to renal failure, mesenteric ischemia and systemic inflammatory response. Thus, is advisable to simplify and shorten this period of the surgery.

In 2008, Lachat et al.^[^^6^^]^ described a technique of sutureless anastomosis by telescoping a stent graft (Viabahn, W. L. Gore & Associates, Flagstaff, AZ) in order to facilitate complex vascular reconstruction in debranching procedures for thoracoabdominal aneurysms. The advantages of this technique are: less dissection of the vessel, lower the ischemic time and simplification of the procedure.

This technique was described solely to distal anastomosis. If also applicable to proximal anastomosis it could enhance its applicability, as in the case of laparoscopic aortic anastomosis.

The objective of this experiment was to test the Viabahn Open Revascularization TEChnique (VORTEC) technique in a swine model, by the construction of an aorto-aortic graft and evaluating the feasibility of the use of the technique in the proximal position. We evaluated the technical success, the time required to perform the anastomosis, the gross macroscopic appearance, and the sealing and the tensile strength of the sutured anastomosis compared to the telescoped anastomosis. The proximal placement of the telescoped graft will, in theory, submit the anastomosis to increased hemodynamic stress in relation to the distal position, which could result in increased rate of migration and leaks. If this model proves successful, the VORTEC technique could be proposed for a broader range of applications.

## METHODS

**Fig. 1:**
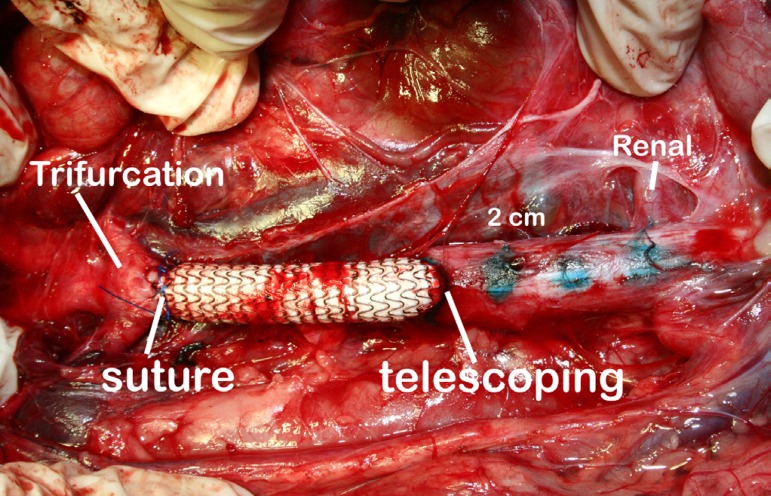
Completed aorto-aortic bypass. The proximal anastomosis is performed using the VORTEC technique and the distal anastomosis in a conventional running polypropylene suture manner.

This study was approved by the Ethical Committee on the Use of Animals of the Institution (protocol 2737-1). The experiment was carried out on eleven female large-white pigs weighting approximately 30 kg. The animals were submitted to general anesthesia with orotracheal intubation, peripheral venous access and invasive arterial pressure monitoring. A median laparotomy was performed exposing the abdominal aorta from the renal arteries to the aortic trifurcation, and the aorta was ligated midway with 2-0 silk. Above the ligature the aorta was punctured with an 18G needle, a 0.035" guidewire was passed to the thoracic aorta and an 18F sheath was introduced 2 cm into the aorta. A 5 mm Viabahn graft was introduced 2 cm into the aorta according to markings previously placed on the sheath. After deploying the endograft, the sheath was removed and the graft was clamped close to its exit from the aortic wall. The distal aorta was transected and a conventional sutured anastomosis was performed between the endograft and the distal aorta with running 5-0 polypropylene (Figure 1).

The patency of the graft was tested by the palpation of the distal pulses and testing with a continuous wave Doppler examination. After completion of the anastomosis, intra-venous adrenaline was injected in order to raise the arterial blood pressure above the normal pressure to check for sealing.

It was annotated the time to perform each anastomosis, the presence of bleeding and/or dislodgment of the endograft.

The animals were divided into two groups. In Group 1, the animals were sacrificed immediately after the operation. In Group 2, the animals were sacrificed after thirty days, meanwhile they were kept at the animal lab's facilities.

After the sacrifice, the anatomic specimen containing the abdominal aorta and the graft was collected for evaluation of a possible displacement of the endograft, presence of stenosis and pseudoaneurysms. In three animals (one in Group 1 and two in Group 2) it was performed a tensile strength test of the anastomosis. This test was done at the Faculty of Agriculture Engineering of the University of Campinas utilizing a press (Otawa Texture Co.), weight cells (Berg Cell), a signal amplifier (Kyowa) and the software DAQWARE, according to previous studies performed on the biomechanics of tendons^[^^7^^]^.

Statistical analysis was performed in the software G-power for the estimation of the sample size and the software IBM's Statistical Package for the Social Sciences for the analysis of the Student t test, for the comparison of time and tensile strength.

## RESULTS

The immediate success of the procedure was 100% as the aorto-aortic graft was completed in all animals. No bleeding was observed from the anastomosis or endograft displacement after the injection of adrenaline to raise the arterial blood pressure after the procedure.

**Fig. 2:**
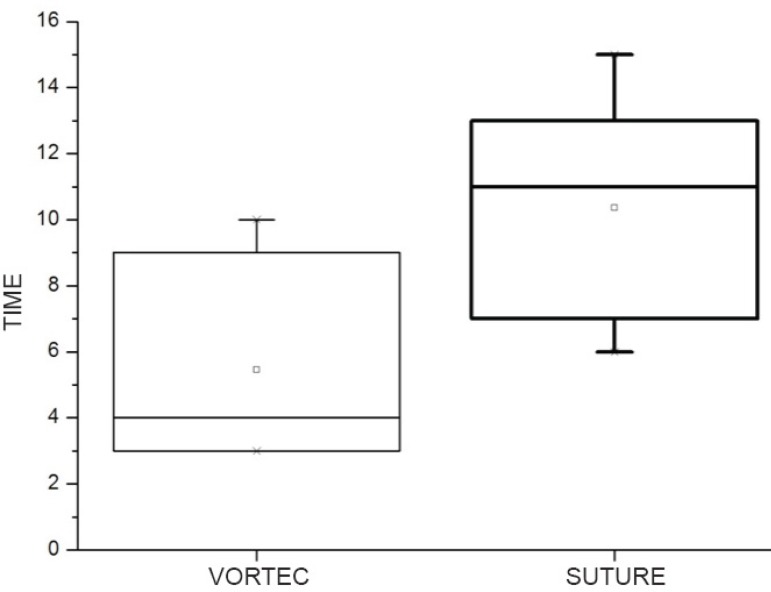
Box plot of “time to complete the anastomosis” in each technique. This graph represents the distribution of the variable. The box represents the percentiles 25, 50 and 75, and the square on the middle, the mean. The x represents percentiles 1 and 99 (minimum and maximum values).

The time to perform the VORTEC anastomosis was shorter compared to the sutured anastomosis (5.4±2.8 min *versus* 10.3±3.4 min, *P*<0.05, Figure 2).

Two animals in Group 2 died on post-operative day one. Necropsy revealed that one animal died from intestinal rupture secondary to intestinal obstruction. In the other animal no cause was identified. A patent graft with no signs of anastomotic rupture was observed in both animals and there was no hemoperitonium.

In the remaining four animals, one graft was found occluded secondary to intimal hyperplasia. The remaining grafts were patent, with no signs of pseudoaneurysms or graft displacement.

**Fig. 3:**
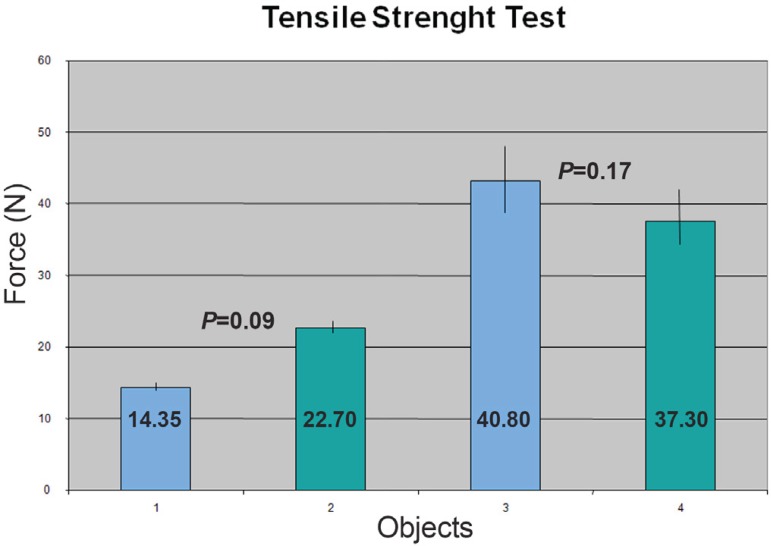
Tensile strength of the anastomosis. In Group 1 (tested immediately after the procedure - objects 1 and 2), there was a bigger difference between the techniques with a higher strength in the sutured anastomosis. In Group 2 (tested 30 days after the procedure – objects 3 and 4), was observed similar tensile strength between the two techniques.

**Fig. 4:**
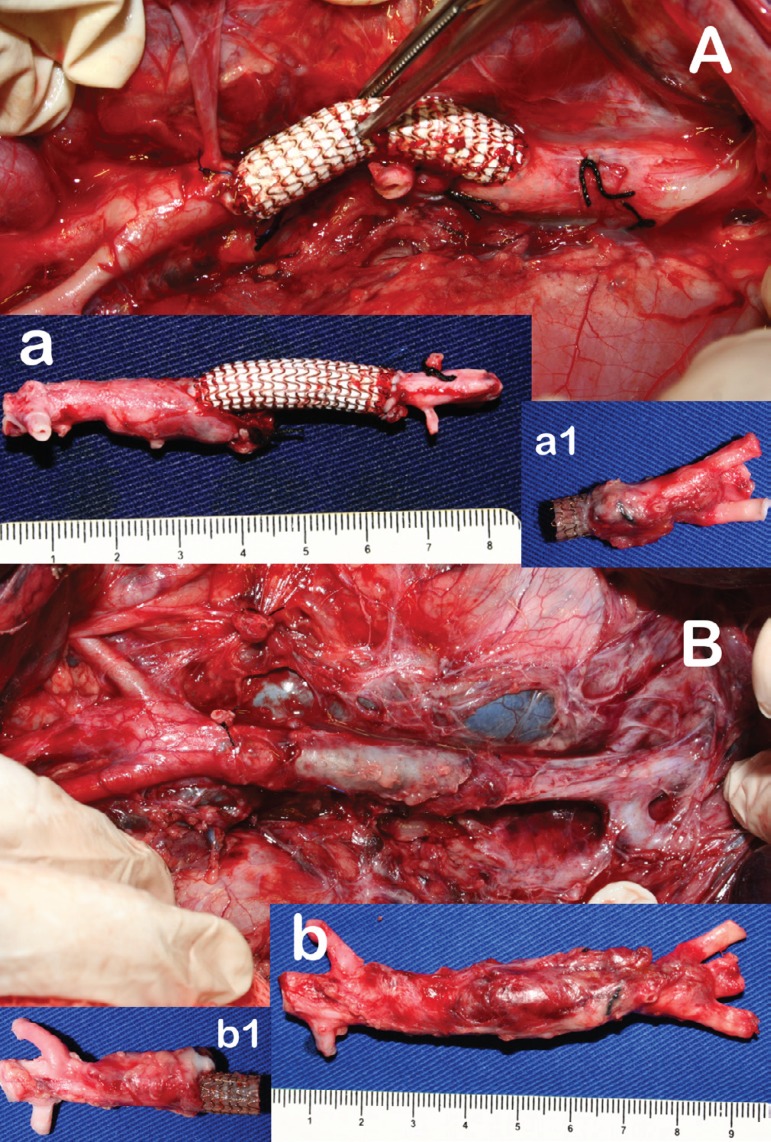
A: graft appearance immediately after implantation. a: after immediate sacrifice. Note that in this picture the graft is positioned inversed in relation to the operative field picture (Group 1). a1: distal sutured anatomosis prepared for tensile strength test (only sufficient scar tissue was removed to allow fixation of the graft to the testing arms. B: graft appearance on post-operative day 30 before explantation. Note the intense scar tissue around the graft. b: after explantation. Note that in this picture the graft is positioned inversed in relation to the operative field picture (Group 2). b1 - proximal telescoped anastomosis prepared for tensile strength test.

The tensile strength test was performed in three animals (Figure 3). On the animal of Group 1 it was found that the sutured anastomosis had a higher strength [22.7 *versus* 14.3 Newton (N), *P*=0.09], as expected, since the proximal anastomosis was anchored only by the radial force of the stent graft. On Group 2, two animals were submitted to the test. It was found no difference between the tensile strength of the sutured and VORTEC anastomosis (37.3 *versus* 40.8 N respectively, *P*=0.17). The increase in tensile strength was probably secondary to the intense fibrous tissue that was found around the operation site (Figure 4).

## DISCUSSION

The VORTEC technique was developed to facilitate the revascularization of the aortic branches during the correction of complex aneurysms. The main advantages would be less dissection of the vessels, lower the ischemic time and simplification of the procedure, decreasing the operative time^[^^6^^]^. This technique is being successfully employed in the treatment of juxta-renal, thoracoabdominal and aortic arch aneurysms^[^^8^^]^. Besides aortic procedures, it has been used in infra-inguinal revascularization with heavily calcified arteries^[^^9^^]^.

In 2012, W.L. Gore & Associates launched on the market the endograft Hybrid, a variation of the Viabahn stent graft, specifically designed to perform this technique because on one end it is a conventional expanded polytetrafluoroethylene graft (ePTFE) graft which facilitates the conventional suture. The other extremity is a stent graft designed for being telescoped into the receptor vessel.

In all these studies the purpose of these grafts was to facilitate the distal anastomosis. This study had the objective to evaluate the performance of this technique as proximal anastomosis, which could broaden the use of this technique.

It was found that the immediate technical success was 100%, the VORTEC technique was faster to perform, as expected, since it requires only the puncture of the vessel under direct visualization, the placement of the sheath and the deployment of the stent graft. Even though the telescoped anastomosis had a smaller immediate tensile strength, after 30 days there was no difference between the two techniques. We believe this is secondary to the formation of fibrous tissue around the anastomosis as observed in the explanted material.

Of course, there are concerns about complications in the long term, such as leakage, kinking and graft occlusion. However, in the short term, this technique applied in the proximal anastomosis confection seems to be very promising.

## CONCLUSION

This study has demonstrated, in a swine model, the feasibility of performing the VORTEC anastomosis in a donor, or proximal position. The technique was faster than the conventional suture technique and after 30 days had a similar tensile strength. This may open the use of this technique in complex debranching procedures, totally telescoped grafts and even facilitating the laparoscopic aortic anastomosis^[^^10^^]^. New hybrid devices may be developed for these applications.

**Table:** 

**Authors' roles & responsibilities**	
LMDF	Conception and design study; realization of operations and/or trials; analysis and/or data interpretation; statistical analysis; manuscript redaction or critical review of its content; final manuscript approval
GBG	Realization of operations and/or trials; manuscript redaction or critical review of its content; final manuscript approval
IMDF	Conception and design study; manuscript redaction or critical review of its content; final manuscript approval
FHM	Manuscript redaction or critical review of its content; final manuscript approval
